# Understanding coach burnout as a relational process: an autoethnographic case from high-performance trampoline

**DOI:** 10.3389/fspor.2026.1815297

**Published:** 2026-05-14

**Authors:** Subaru Shintani, Masaaki Sugita, Jun Mizushima

**Affiliations:** 1 Faculty of Health and Sports Sciences, Toyo University, Tokyo, Japan; 2 Faculty of Sport Sciences, Nippon Sport Science University, Tokyo, Japan

**Keywords:** autoethnography, coach burnout, coaching identity, high-performance sport, relational dynamics

## Abstract

This study explored how burnout was experienced, interpreted, and responded to by a trampoline coach working in a high-performance context. Adopting an autoethnographic case study design, the first author reflected on his own burnout experience through retrospective dialogic interviews and sustained reflexive engagement across an Olympic cycle. Rather than treating burnout as a discrete endpoint or acute breakdown, the analysis traced how relational responsibility, organisational expectation, contested coaching roles, and political scrutiny accumulated over time. Findings show how commitment initially experienced as meaningful became increasingly binding, narrowing interpretive flexibility and reducing perceived agency. Burnout was experienced not simply as exhaustion, but as a growing mismatch between expanding responsibility and a diminishing capacity to reconcile competing expectations. Symptoms of strain emerged gradually and were normalised within ongoing performance preparation, delaying recognition of deterioration. A disruption of routine structures during the pandemic did not initiate burnout but made the depth of accumulated strain visible. Later responses were characterised not by decisive recovery, but by partial withdrawal, boundary renegotiation, and ongoing reconfiguration of professional identity. By conceptualising burnout as a temporal and relational process of internalised responsibility within high-performance sport systems, this study contributes to a more contextually grounded understanding of coach wellbeing. The findings highlight the importance of addressing not only individual coping strategies, but also the organisational and relational conditions through which responsibility is structured and sustained in elite sport environments.

## 1 Introduction

Coach burnout has become an increasingly prominent concern within sport coaching research, particularly in relation to high-performance sport contexts. Existing studies have consistently shown that coaches operating in such environments are exposed to multiple and often competing demands, including performance expectations, extensive time commitments, organisational pressures, and complex interpersonal relationships (1–3). These demands place coaches at heightened risk of burnout, commonly conceptualised through dimensions such as emotional exhaustion, depersonalisation, and reduced personal accomplishment (1), drawing on influential models that frame burnout as a psychological syndrome experienced at the individual level (4). While such models have been foundational in advancing burnout research, they offer limited insight into how burnout unfolds relationally and contextually over time within specific high-performance environments. Coach burnout has been linked to negative outcomes not only for coaches themselves, but also for athlete development, coaching effectiveness, and organisational sustainability (3).

Despite the growing body of literature on coach burnout, much of this work has been dominated by quantitative approaches that focus on prevalence, correlates, and predictors of burnout symptoms [e.g., (2, 5, 6)]. A scoping review by Olusoga et al. (2) confirmed that the vast majority of research (84.45%) remains quantitative, with 80% employing cross-sectional designs that struggle to reflect the enduring and dynamic nature of the burnout experience. This echoes earlier concerns raised in the systematic review by Goodger et al. (7), which synthesised 58 studies and identified key psychological correlates such as motivational loss and situational factors like role conflict, while noting that the reliance on group-comparison designs means the actual burnout experience of affected individuals often becomes “lost in the crowd”.

Addressing this gap, qualitative researchers have explored the lived experience of burnout. For example, Coakley (8) used a sociological perspective to show how young individuals can experience identity foreclosure and a loss of autonomy within sport organisations. Similarly, Lundkvist et al. (9) identified two distinct qualitative profiles: a “performance culture” profile, where identity is tied to results and symptoms manifest as bitterness and cynicism, and a “life situation” profile, where an overwhelming total workload leads to profound physical exhaustion. These qualitative insights reveal how pressures accumulate over time and demonstrate that recovery requires either reconfiguring one's professional role within the sport or rebalancing the total life situation.

The need for such contextualised understanding is especially salient in high-performance sport, where Goodger et al. (7) noted a “notable absence” of elite-level samples in the literature, Olusoga et al. (2) revealed that this gap persists, with high-performance coaches accounting for only 15.6% of research samples. In this environment, coaching success is frequently judged through competitive outcomes and public performance indicators (10). Prior research suggests that high-performance coaches often operate in environments characterised by heightened accountability, organisational politics, and role ambiguity (11, 12). Paradoxically, these pressures may intensify even during periods of competitive success, when expectations escalate, and responsibilities expand (12). However, relatively little research has explored how high-performance coaches themselves make sense of burnout in moments when outward indicators of performance appear positive (13). Understanding burnout as a lived and contextualised experience is therefore essential for advancing more nuanced and practically meaningful insights into coach wellbeing in high-performance sport.

Narrative and interpretive approaches are particularly suited to addressing these gaps by foregrounding coaches’ voices and the subjective complexities of identity and emotion (14, 15). Within this broader qualitative tradition, autoethnography has emerged as a method that enables researchers to draw on personal experience to illuminate coaching work as it is lived, while situating that experience within relevant social, cultural, and organisational contexts (16). In sport and coaching research, autoethnographic studies have been used to illuminate issues such as identity negotiation, and the often-invisible dimensions of coaching practice (17, 18). Nevertheless, autoethnography remains underutilised in the specific examination of coach burnout, particularly within high-performance environments.

Therefore, the purpose of the present study is to explore how burnout was experienced, interpreted, and responded to by a trampoline coach working in a high-performance context. Adopting an autoethnographic case study design, the first author reflects on his own burnout experience through retrospective interviews and ongoing reflexive dialogue with a critical friend. By focusing on a single, in-depth case, this study seeks not only to illuminate the cumulative and contextual nature of coach burnout, but also to examine how escalating patterns of relational responsibility and organisational expectation shaped its gradual development. In doing so, the study aims to contribute to a more process-oriented and relational understanding of coach wellbeing in high-performance sport and to offer practical insights for researchers, coach developers, and sporting organisations seeking to support coaches in demanding environments.

## 2 Methods

### 2.1 Research design

This study employed an autoethnographic case study design to explore burnout as it was lived within the everyday relational and organisational complexities of high-performance coaching. Specifically, it adopted an interpretive case study design (19), which can be understood as an instrumental case study (20). In this approach, the first author's specific coaching career is not treated as a unique or isolated bounded case, but rather as a contextually embedded site through which broader relational and organisational processes of burnout can be analytically illuminated. This positioning aligns with perspectives that view case studies as offering theoretical insight through in-depth contextual analysis (21, 22). Autoethnography was selected not to offer representative claims, but to provide an in-depth account of these complex processes that may remain obscured in more decontextualised designs (23).

This study is grounded in a social-constructionist orientation, assuming that meanings of burnout are not objective, fixed entities to be discovered, but are discursively produced and co-constructed through situated interpretation. Within this framework, knowledge is regarded as subjective and fluid, emerging through the reflexive interaction between the researcher's past lived experience and present reflection. Accordingly, the account presented here is viewed as a co-constructed narrative, acknowledging that all knowledge is partial and relationally produced (15).

This epistemological positioning informed the adoption of a dialogic approach to analysis. Rather than treating reflection as a solitary process, the involvement of a critical friend (the last author) introduced a degree of analytical distance that enabled questioning, reframing, and alternative interpretation of the author's experiences. Through iterative interviews and sustained reflexive dialogue, this process supported the development of a more critically examined and analytically robust account, helping to situate personal experience within broader organisational and relational contexts.

### 2.2 Case description

This case was situated within a high-performance sport context characterised by sustained performance demands, organisational accountability, and close interdependence among athletes, coaches, and support staff across the Olympic qualification cycle, within the sport of trampoline gymnastics.

Prior to coaching, I (the first author) competed internationally as a high-performance trampoline gymnast, achieving world-level success in junior and youth categories. I retired from competition during my undergraduate studies and transitioned relatively early into coaching, a trajectory that shaped my professional identity and engagement with high-performance environments.

I coached across multiple performance levels (high school, university, and senior athletes). For a period during an Olympic qualification cycle, I served as a national-level coach within the sport's high-performance system. During this period, I held formal responsibilities within the national coaching structure while simultaneously acting as the personal coach of individual athletes, requiring ongoing negotiation of competing roles and accountabilities.

This case was selected as an information rich instance (20), in which multiple sources of pressure, including performance expectations, organisational accountability, and relational responsibilities, converged within a single coaching role. The overlap between institutional responsibilities and personal coaching relationships created conditions in which tensions around legitimacy, responsibility, and role clarity could be examined more explicitly. As such, this case provides a contextually embedded site for examining how burnout emerges not only from workload, but from the relational and organisational dynamics that structure high-performance sport systems.

### 2.3 Data generation

Data were generated through three retrospective semi-structured interviews conducted between the first and last authors. The interviews lasted 60, 69, and 71 min, respectively, and focused on my coaching career trajectory, experiences of escalating strain and burnout, critical incidents, and subsequent responses. All interviews were audio-recorded and transcribed verbatim. The interview questions were developed in an iterative and flexible manner, guided by key thematic areas derived from the research purpose, including coaching trajectory, experiences of strain, critical incidents, and responses to burnout.

Following transcription, we engaged in ongoing reflexive dialogue between the first author and a critical friend, who challenged assumptions and prompted alternative interpretations. These exchanges were loosely structured around revisiting key moments from the interviews, exploring alternative meanings, and interrogating taken-for-granted interpretations, while remaining responsive to emergent lines of inquiry. These conversations were audio-recorded and supported by collaboratively produced, date-stamped reflexive memos. The memos documented emerging interpretations, emotional responses, points of tension, and shifts in meaning over time. This dialogic material was treated as an extension of data generation rather than as a discrete analytic phase, consistent with dialogic approaches to autoethnography.

To support accuracy of recall and deepen contextual understanding, I also consulted contemporaneous artefacts, including training plans, competition schedules, and selected written communications (e.g., emails and athlete- and team-related communications). These materials were not analysed as standalone data sources, as the primary analytic focus was on the interpretive reconstruction of lived experience rather than on document analysis. Instead, they were used to situate narrated experiences within their organisational and temporal contexts and to prompt further reflection during interviews and dialogue. All identifying and potentially deducible information within these artefacts was removed or deliberately generalised to minimise the risk of deductive disclosure.

### 2.4 Data analysis

Analysis was conducted through iterative, dialogic reconstruction of lived experience rather than formal thematic coding. Consistent with analytic autoethnography (24) and contemporary autoethnographic practice (25), knowledge was approached as situated and relationally co-constructed through reflexive engagement with experience and dialogue, rather than as categories to be discovered.

The analytic process involved repeated engagement with interview transcripts, reflexive memos, and artefacts across the Olympic cycle. Instead of seeking categorical saturation, attention focused on tracing shifts in responsibility, identity positioning, relational tension, and embodied strain over time. Analysis centred on identifying moments of intensification, rupture, reorientation, and gradual escalation within the high-performance context, consistent with narrative and process-sensitive qualitative approaches that emphasise temporal unfolding (26).

Interpretive shifts were identified through iterative comparison across transcripts and reflexive memos, particularly where changes in how experiences were described, evaluated, or emotionally expressed became evident. Analytic decisions were documented through date-stamped reflexive memos, which captured emerging interpretations, points of uncertainty, and moments where initial understandings were revised.

A phase-based process representation was constructed through collaborative dialogue and temporal mapping of critical events. The four phases were analytically generated from clusters of interpretive shifts identified in the data, particularly where responsibility attribution, emotional tone, and perceived agency reorganised across transcripts and memos. Phase boundaries were delineated by identifying points at which these patterns shifted in a sustained and consistent manner across multiple data sources, rather than in response to single events. These phases were not treated as predetermined stages, but as representations of evolving relational and organisational dynamics across the Olympic cycle.

Throughout the study, the last author acted as a critical friend, questioning assumptions and offering alternative interpretations (27). For example, I initially framed my continued acceptance of expanding responsibilities as professional commitment. The critical friend asked whether this reflected deliberate choice or a gradual narrowing of perceived alternatives. Revisiting the data revealed that responsibility had expanded alongside reduced influence over key decisions. This exchange contributed to reframing burnout not simply as overwork, but as evolving responsibility without corresponding control.

In another instance, I initially understood my role as involving a high degree of autonomy. However, revisiting the data showed that this autonomy was accompanied by increasing self-blame when I could not manage competing expectations. This led to a shift in interpretation, from viewing autonomy as a source of control to understanding it as a condition in which responsibility increased without sufficient influence over outcomes. This iterative comparison strengthened the alignment between the data and the interpretive claims.

Analytic rigour was pursued in line with qualitative criteria emphasising reflexivity, transparency, and coherence (28). Reflexivity was maintained through ongoing critical engagement with the data, allowing assumptions to be questioned and interpretations to be continually refined. Transparency was achieved by clearly documenting the processes of data generation and analysis, enabling readers to follow how interpretations were developed. Coherence was supported through careful alignment between experiential material, and interpretive claims, and the study's conceptual framing.

### 2.5 Ethical considerations

This study received ethical approval based on utilitarianism and aspirational principles (15). Consistent with prior autoethnographic research in sport coaching [e.g., (18, 29)], formal consent from other individuals was not required, as the study focused on the first author's own experiences. Nevertheless, careful attention was paid to ethical relationality and confidentiality. Individuals who were directly implicated in narrated events were informed of the study's focus where appropriate. Identifying details relating to organisations, individuals, and specific competitions were omitted or deliberately generalised. Where temporality or events were analytically relevant, non-identifying descriptors (e.g., “an international competition” and “the Olympic qualification cycle”) and broader timeframes were used.

Given the small and tightly networked nature of high-performance trampoline, particular attention was paid to the risk of deductive disclosure. In addition to removing names and specific identifiers, certain temporal markers and contextual details were generalised or slightly blurred where necessary to reduce traceability. Sensitive experiences were reported from the perspective of the first author's interpretation rather than as evaluations of others. These decisions were guided by principles of relational ethics, prioritising the minimisation of potential harm within a close-knit performance environment.

## 3 Results

The results are organised into four analytically constructed phases tracing the development of burnout across the Olympic cycle. Rather than representing a discrete breakdown, burnout unfolded as a cumulative process shaped by expanding responsibility, organisational ambiguity, and relational strain. The following sections describe each phase in detail.

### 3.1 Phase 1: an unplanned entry into the national system and the emergence of a reform-oriented commitment

My entry into the national coaching system was neither strategic nor driven by coaching achievements. At that point, I had no notable record as a coach, and I had never actively sought a role within the national team structure. Instead, my involvement began incidentally, through a request to deliver an anti-doping lecture at a national training camp during my early doctoral studies in pharmacy.

I attended the training camp primarily in the role of an educator rather than a coach. The athletes present were mostly familiar to me; many were only two or three years younger, and several had been teammates or acquaintances from earlier stages of my athletic career. As a result, my initial presence was marked by ease rather than friction. Without any formal transition, I was gradually invited to observe training sessions. What initially took the form of casual requests such as “just have a look at today's practice” or “tell us what you think” slowly developed into more sustained involvement. These invitations were often made directly by a senior figure within the performance system, who was also affiliated with the university environment where I had previously studied. In this tightly connected and informal sporting environment, personal relationships facilitated access more than formal credentials.

Importantly, I did not step into this space with the intention of becoming a national coach. I never consciously decided, “I will be a coach here”. Rather, as I later reflected, I entered without making a clear commitment, and only retrospectively did I realise that I had assumed the role. It felt less like choosing to become a coach and more like becoming one by default, through a series of small, unresisted steps.

As I spent more time within the national training environment, I began to hear athletes articulate frustrations that closely resembled my own experiences as a former junior athlete. Despite being labelled as “national training camps”, the objectives and intentions of these camps were often unclear. Training lacked coherence at the team level, and dissatisfaction toward coaching practices and organisational decision-making was common. What struck me most was that many of the coaches occupying key positions were the same individuals who had been present during my own youth career. From my perspective, little appeared to have changed.

It was within this context that a sense of responsibility began to emerge. I started to believe that by positioning myself closer to the centre of the system, and by working under the performance director, I might be able to address the very issues that had frustrated me as an athlete. Improving the environment for athletes, correcting structural ambiguities, and creating conditions that allowed athletes to train with clarity and purpose became intertwined with my growing involvement. These aspirations aligned naturally with performance goals, including Olympic qualification, and gradually deepened my engagement.

A critical turning point occurred when one athlete directly asked me to coach them personally. Entering into a contractual relationship with an individual athlete fundamentally altered my relationship to the national system. From that moment, the quality of the national training environment was no longer an abstract organisational concern; it directly affected the performance and wellbeing of an athlete for whom I felt personally responsible. If the national system was dysfunctional, it would inevitably compromise that athlete's development. This realisation intensified my commitment and solidified my presence within the national structure.

At this stage, my involvement did not feel burdensome. On the contrary, it was experienced as meaningful and even rewarding. I perceived myself as confronting problems I genuinely wanted to solve. Addressing institutional shortcomings, supporting athletes, and contributing to performance improvement felt aligned with both my personal values and professional aspirations. Looking back, this phase was characterised less by strain than by immersion—an earnest and increasingly invested attempt to make the system better from within.

### 3.2 Phase 2: the crystallisation of burnout through contested roles, political scrutiny, and the erosion of self

This phase marks the point at which burnout became explicit and recognisable, not as a sudden collapse, but as the accumulation of unresolved tensions that reached a critical threshold. In my recollection, this process began gradually during the latter phase of the Olympic qualification cycle and intensified toward a major international championship.

Looking back, this period was characterised by a growing sense that “everything started to pile up”. During the later stages of the Olympic qualification cycle, my involvement within the federation increasingly felt burdensome. Responsibilities expanded, informal expectations accumulated, and I became aware that my position was being viewed critically by some within the organisational environment. These organisational and relational pressures did not develop independently. Rather, they overlapped and reinforced one another, producing a sense of compression in which previously manageable stressors became inseparable. This accumulation reflected not a single organisational failure but the gradual intensification of role ambiguity, relational tension, and accountability pressures characteristic of high-performance coaching environments.

A major international competition functioned as a moment of condensation rather than a singular trigger. By that point, multiple unresolved tensions had converged, revealing rather than causing the burnout process. It was during this period that I became certain this would be my final involvement as a national coach. This decision did not arise from a single incident, but from the recognition that the problems I was carrying could no longer be disentangled or negotiated within the existing system.

At the centre of this phase was the problematisation of my dual role. Formally, I was positioned as a national team coach within the federation. Simultaneously, I was acting as the personal coach of an athlete competing at a major international event and situated on the margins of Olympic qualification. In principle, such dual roles are often framed as problematic within high-performance sport systems, where separation between national and personal coaching is emphasised to avoid conflicts of interest, despite such arrangements remaining common in practice. In this context, however, chronic understaffing and resource limitations meant that such arrangements were tacitly accepted. During this period, the contradiction between normative governance ideals and structural realities became explicitly problematised.

Political scrutiny intensified around my position. Questions were raised regarding the legitimacy of my presence at the competition, and organisational decisions were made that required me to demonstrate visible separation from the athlete I personally coached. This arrangement created a profound sense of disorientation. I described reaching a point where I could no longer identify what I was meant to protect, or whom I was meant to be acting for. Managing competing expectations across organisational authorities, the coaching community, and the athlete themselves gradually came to feel meaningless. Rather than working toward resolution, my attention shifted toward the sense that this situation was “just a hassle”, and more troublingly, “a waste of life”.

Crucially, this was also the moment at which I recognised my own emotional withdrawal. Once this disengagement set in, I was no longer fully confronting the situation. The decision to step away from national coaching did not emerge solely from anger or frustration, but from the recognition that my commitment had already eroded at a deeper level.

Alongside this, feelings of powerlessness intensified. Although I intellectually understood that the influence of a national coach in an individual sport is structurally limited, I experienced growing frustration at having entered this environment without fully grasping those constraints. The presence of multiple coaches, each operating from different value systems and coaching beliefs, further complicated the possibility of coherent action. Within this context, attempting to respond simultaneously to athletes’ expectations, fellow coaches’ beliefs, and organisational imperatives gradually became unsustainable.

As these demands accumulated, I described a process of self-erasure. In attempting to meet competing expectations, I found myself performing different versions of myself for different audiences. Over time, this performance displaced any coherent sense of identity. The dominant emotional outcome was not anxiety or anger, but emptiness. I experienced a growing sense of nihilism in which I no longer knew why I was doing this work or for whom.

This emotional depletion coexisted with apparent formal autonomy. I perceived myself as having substantial discretion over training structures and selection-related processes. Rather than empowering me, this autonomy intensified self-blame. I repeatedly questioned how I could have failed to reconcile competing interests despite being afforded such freedom. The contradiction between perceived autonomy and limited capacity to effect meaningful change deepened my sense of personal inadequacy.

This phase represents the point at which burnout became embodied as a loss of meaning. It was not exhaustion or conflict alone, but the realisation that no configuration of action could satisfy competing demands without erasing the self. The major international event did not cause burnout; it revealed it. By the end of this phase, disengagement was no longer a possibility to be considered. It had already begun.

### 3.3 Phase 3: embodied breakdown, affective flattening, and the collapse of functional coping

This phase marked the point at which burnout became unmistakable in my body, cognition, and everyday functioning. Rather than a single dramatic event, it unfolded as a progression. Initially familiar psychological stress symptoms appeared manageable, then accelerated into a self-reinforcing cycle, and eventually culminated in a state where I could no longer function reliably as a person.

The earliest and clearest signal was insomnia. Once sleep began to deteriorate, I experienced what I described as a negative spiral that “kept turning”. Persistent sleep disruption was followed by a widening cluster of symptoms that, in retrospect, resembled what is commonly associated with psychological collapse. These included palpitations, migraine, and episodes of stomach cramping. Cognitive capacity also shifted. I did not stop reading altogether; rather, text could be visually processed but no longer entered my mind in a meaningful way. Sound and light became overwhelming. I withdrew socially, became increasingly homebound, and experienced pervasive fatigue in which my body would not move. As my relational world narrowed, I cut off contact, reacted more aggressively than intended, and lost interest in people, tasks, and activities that had previously mattered. Throughout this period, suicidal ideation was present in the background, expressed in recurring thoughts such as “I want to die” or “I do not want to do anything anymore”.

At the time, however, I did not fully recognise these experiences as symptoms. I described myself as “running”, and while running, I did not stop to interpret what was happening. Looking back, I noticed more frequent cold-like illnesses and an increased occurrence of headaches, but these changes were not treated as warnings. The major international competition ended, still, the Olympic qualification process continued. Later in the season, I attended largely out of inertia. Having already committed, I felt compelled to continue.

Shortly thereafter, the pandemic-related suspension of international competitions exposed a contradiction I could no longer ignore. While others framed the postponement as an additional opportunity, my response was visceral relief. I remember thinking, from the bottom of my heart, “Yes. It's gone”. Rather than disappointment, I felt joy at the suspension of national activities. This reaction revealed something unsettling—not only was I exhausted, but a part of me no longer wished to be involved at all.

During the subsequent lockdown, I spent approximately three and a half months in a markedly different rhythm of life. National activities ceased, training centres closed, and the athlete I personally coached relocated with me to a rural club environment. The Olympic pathway was no longer viable at that time, and the absence of national obligations created a space free from constant self-monitoring, explanation, and political navigation. I described this period as deeply comfortable, not simply because I was resting, but because the conditions sustaining strain were temporarily removed. Looking back, much of this broader period is not readily accessible in memory. Outside the lockdown months, recollections often remain fragmented and temporally blurred, as if parts of the experience have “dropped out”. I noticed that detailed scenes tended to return only through dialogue, when talking prompted specific moments to resurface.

As the resumption approached, I experienced an abrupt internal collapse. I recalled thinking, “It is going to start again”, followed not by a sense that something had snapped, but by the realisation that whatever had previously been keeping me going had already burned out. For some time, I had been forcing myself to reignite motivation through effort and obligation. At that moment, however, no amount of effort could spark it again. What had been sustained through sheer momentum could no longer be reanimated. Anxiety intensified, and at the same time I felt completely immobilised. In simple terms, I was confronted with the overwhelming sense that, no matter how hard I tried, I could no longer move. Symptoms intensified rapidly and became impossible to ignore.

Approximately one month later, I sought medical help. Although I had sensed that earlier intervention was necessary, I had been unable to act until the severity of my condition became undeniable. At a psychosomatic clinic, I was informed that I was in a depressive state and warned that, if untreated, the risk of self-harm was significant. From that point onward, regular clinical care became part of my life.

These changes were not only internal. They became visible to the athlete I coached. Although I attempted to appear unchanged, my behaviour shifted. I began taking week-long breaks and delegating sessions, and such absences became more frequent. Communication boundaries narrowed. Where I had once responded at any hour and adapted schedules flexibly, I imposed fixed time limits and reduced availability. I carried two phones and separated accounts to manage demands. Planning capacity also deteriorated. Rather than engaging in forward-looking discussions about future Olympic cycles, I relied on short-term slogans such as “let's just do what we can now”, not as a strategic choice but because I could no longer sustain projection into the future. In retrospect, this represented a loss of responsibility. From the athlete's perspective, I had said “let's do this together”, yet I could no longer inhabit that commitment.

Even after withdrawing from national travel, disengagement remained partial. I stopped attending tours, aware that this would be interpreted negatively; by that point, I no longer had the emotional capacity to care. At the same time, residual organisational expectations persisted. I agreed to minimal symbolic participation in pre-Olympic activities, appearing briefly in training camps “for form's sake”, despite believing my presence was unnecessary. Ultimately, I did not accompany the team as an Olympic coach, although I remained formally listed in a reduced capacity.

Across this period, my understanding of burnout shifted. I came to interpret burnout not as a moment, but as a process. The major international competition marked the point at which I realised my interest in the sport may already have been gone. Over subsequent months, that realisation hardened into certainty, followed by depression. The clearest evidence was emotional. Despite objectively extraordinary success, including strong national results and podium performances by the athlete I personally coached, nothing in me moved. I felt no joy, only strain and a desire for it to end. The pandemic did not create this condition; it revealed it. During the months without national obligations, I experienced relief and even enjoyment. When it became necessary to re-enter the system, I discovered that I could not. What had previously been kept alive through repeated effort now refused to ignite at all. At that point, burnout ceased to be a professional concern and became a whole-life breakdown, shaping my relationships, my routines, and my capacity to function.

### 3.4 Phase 4: distancing attempts, constrained support, and an unfinished reconfiguration of coaching identity

After the symptoms described in Phase 3 became unavoidable, my response was not recovery in any straightforward sense, but an attempt to create distance from what was harming me. Support was present at an interpersonal level, though it remained insufficient to produce structural change. Because I occupied a position close to senior leadership within the performance structure, I was able to consult regularly. There was space for conversation, and my difficulties were acknowledged. However, these discussions consistently ended at the same point. The situation was understood as difficult but unavoidable. In high-performance trampoline, where resources were limited, constraints were treated as fixed conditions rather than problems that could be altered. Over time, these conversations came to feel less like problem-solving and more like shared endurance. Pain could be named, but nothing fundamentally changed.

Outside that small professional circle, the severity of my condition was largely invisible. For those I did not speak to directly, it likely appeared that I had simply stepped back by choice. Withdrawal was visible, but exhaustion was not. Friends and family did listen to me, and I did tell them that I was struggling and attending a clinic. Even there, I gradually recognised a limit. Talking provided momentary relief but no resolution. I came to feel that repeatedly describing my suffering was not moving anything forward. The problem was not a lack of care, but the absence of any pathway through which care could alter the conditions producing distress.

The primary strategy I adopted was withdrawal and selective disengagement. At the national level, this was decisive. I withdrew from the national team and made it clear that I would not be involved in the upcoming Olympic Games. In that sense, I did step away. However, distancing was incomplete. I continued coaching my personal athlete, and as long as I did so, I remained connected to the high-performance system. The distance I created was therefore partial and temporary. I began to take short breaks, delegating responsibilities for limited periods. At the time, these pauses felt helpful. They provided relief and the impression of stepping back.

With hindsight, I can see that this distance was superficial. Relief was short-lived, and I always returned to the same demands. More decisive separation might have been necessary, but at that moment my perspective was narrow and my capacity to act severely diminished. Even though continuing often felt unbearable, it still felt easier than making a radical change. Action required energy and clarity that I no longer had.

During this period, my understanding of myself also shifted. One of the strongest realisations was that I had not known my own limits. In my late twenties, I assumed that I would not reach a breaking point. I overestimated my capacity to endure and underestimated my vulnerability. At the time, I did not even have language for what was happening. I did not know the term burnout. I only knew that my life was no longer functioning. Being told at a psychosomatic clinic that I was in a depressive state marked a turning point. It did not resolve the situation, but it made clear that continuing unchanged was no longer possible.

Even now, I would not describe this phase as recovery. Trampoline still carries emotional weight, and contact with the sport can evoke strain. What did begin to change was my relationship to coaching itself. Previously, my identity had been almost entirely organised around producing results. Gradually, I began to see performance as one dimension of sport rather than its defining purpose. Responsibility came to be understood differently, with greater concern for how athletes exit high-performance sport and move into the next stage of life.

This reorientation was supported by my transition into a university-based role, where sport was situated within broader educational and developmental purposes rather than treated as all-encompassing. In that context, I could begin to imagine forms of coaching engagement that did not require complete self-erasure. Still, this phase does not end with resolution. The strain has not disappeared. What changed was my position in relation to it. Rather than recovery, this phase represents an unfinished reconfiguration—an ongoing attempt to construct a livable coaching identity while accepting that some aspects of the experience remain unresolved.

## 4 Discussion

This study explored how burnout was experienced, interpreted, and responded to within a high-performance trampoline coaching context. Rather than treating burnout as a discrete breakdown, the analysis shows how it developed gradually within the everyday relational and organisational conditions of coaching work. By tracing shifts in responsibility, legitimacy, and professional identity across the Olympic cycle, the study conceptualises burnout as a temporally unfolding process marked by relational entanglement and narrowing agency. Burnout was therefore not experienced simply as exhaustion, but as a progressive mismatch between expanding responsibility and a diminishing capacity to reconcile competing organisational, relational, and performance expectations.

The present case can be situated within existing coach burnout literature, which identifies organisational, contextual, interpersonal, and intrapersonal stressors as key contributors (2, 5). Previous work has linked burnout in high-performance coaches to workload, work–home interference, reduced recovery, lower need satisfaction, and more controlled forms of motivation (2, 5). The current findings extend this literature by illustrating how these factors may converge over time as a growing imbalance between responsibility and the capacity to reconcile competing expectations. In this sense, the case aligns with worklife models that conceptualise burnout as arising from mismatches between individuals and their work environment, including workload, control, reward, community, fairness, and values (4).

A central insight concerns how early commitment to improving athlete conditions, grounded in a strong sense of personal responsibility, was initially experienced as meaningful and purposeful. Over time, however, this same commitment was reinterpreted as binding and inescapable, illustrating how vulnerability emerged not from workload alone, but from deepening identification with the system. In this process, burnout was experienced as a gradual narrowing of interpretive flexibility and perceived agency. Previous research has noted that high-performance coaches often operate with significant responsibility relative to their formal authority (11). In this case, responsibility was experienced not only as structurally positioned, but as relationally enacted and sustained in daily coaching interactions.

Organisational ambiguity, political scrutiny, and contested roles were not encountered merely as structural stressors; they were lived as relational tensions that unsettled identity and disrupted meaning. In an aesthetically judged sport such as trampoline, where performance evaluation is highly visible and closely tied to perceptions of coach legitimacy, these tensions were not diffused by success but, at times, intensified by it. This case may be especially relevant to judged high-performance sport contexts, where accountability and evaluation are intensified, and where role tensions may be further amplified.

Over time, these pressures became embodied. Symptoms such as insomnia, cognitive impairment, emotional withdrawal, and depressive thoughts emerged gradually and were initially normalised or overlooked within the demands of ongoing performance preparation. This trajectory resonates with existing burnout research (4), while drawing attention to how deterioration can remain normalised within the rhythms of ongoing preparation and competition. What is notable in this case is not simply the presence of these symptoms, but the extended period during which their significance remained unrecognised. Continued engagement in competition and preparation created conditions in which deterioration could be interpreted as manageable strain rather than as warning signs of collapse.

In this case, a clinical diagnosis of a depressive state was made during the later stages of the burnout process. While burnout may co-occur with depressive symptoms, it is understood as a work-related phenomenon rather than a clinical disorder (30). Accordingly, we interpret this case as burnout-related distress within a high-performance coaching role, rather than depression per se.

The interruption of routine structures during the pandemic did not initiate burnout; rather, it made its depth visible. The sudden suspension of national activities disrupted the rhythm that had previously sustained continued engagement, and in that pause, the absence of motivation became unmistakable. This pattern is consistent with narrative and interpretive research suggesting that exhaustion and identity disruption often become perceptible only when habitual practices are interrupted (31, 32). At the same time, the present case appears to add something not yet well developed in the coach burnout literature. Existing reviews have noted both the scarcity of research with high-performance coaches and the limited attention given to burnout as an unfolding, recognisable process, particularly in relation to withdrawal and recovery (2). To our knowledge, coach burnout research has rarely examined how previously normalised depletion becomes visible only when routine high-performance practices are disrupted. In this respect, the current study offers a processual account of how burnout was not immediately named or recognised, but became intelligible through interruption, reflection, and retrospective reconstruction.

At this stage, qualities typically valorised in high-performance sport, including effort, endurance, and a strong professional identity, no longer operated as protective resources. Instead of buffering strain, they appeared to sustain continued engagement beyond healthy limits. Perseverance, which had previously enabled functioning under pressure, became a mechanism through which exposure to stress was extended rather than alleviated. This pattern aligns with burnout research indicating that when sustained demands exceed available resources, exhaustion and disengagement are more likely (4). In such conditions, commitment does not necessarily prevent burnout; it may deepen it by making withdrawal more difficult.

Individual coping strategies proved insufficient, and clinical support became necessary. Although organisational leaders, colleagues, friends, and family offered care and validation, these forms of support did not alter the conditions generating strain. The primary sources of distress were embedded in organisational arrangements and relational expectations that extended beyond any single interaction. This case therefore illustrates how, in resource-constrained high-performance sport environments, support may be interpersonal while strain remains structural. Previous research suggests that such environments often rely heavily on individual commitment, personal responsibility, and informal problem-solving rather than robust systemic safeguards (3, 11). Recent syntheses of coach mental health research further highlight how emotionally demanding and politically sensitive relational environments compound these risks, particularly where formal support mechanisms are limited (1).

In light of these findings, this single retrospective autoethnographic case offers insight into high-performance sport organisations. First, coach wellbeing may be compromised when responsibility expands without corresponding authority, role clarity, or meaningful feedback. Second, organisations should not assume that coach distress is best understood as an individual coping deficit; organisational stressors such as role ambiguity, conflict, feeling undervalued, and limited life balance are repeatedly linked to poorer coach mental health. Third, support systems should include proactive monitoring, psychologically safe help-seeking pathways, mental health literacy, and clear rehabilitation and reintegration processes for coaches experiencing mental ill-health. In this regard, the present case suggests that burnout prevention in high-performance sport is a matter of organisational design as much as individual resilience.

Rather than marking a clear recovery, the later period was characterised by distancing and reconfiguration. Withdrawal from national-level coaching reduced exposure to the most intense pressures, but disengagement remained partial and constrained by livelihood, responsibility to athletes, and diminished agency. Competitive success no longer functioned as the primary organising value. Instead, coaching came to be understood as one dimension of a broader life trajectory, both for the athlete and for myself. This shift involved not a restoration of a prior coaching self, but a recalibration of professional identity. Responsibility was not relinquished, but enacted within more explicit boundaries. In this sense, recovery was less about symptom reduction and more about reconfiguring the conditions under which commitment could be sustained.

More broadly, this case suggests three transferable insights for future research and policy: that burnout risk intensifies when responsibility expands faster than the capacity to reconcile competing expectations, that relational support cannot compensate indefinitely for chronic organisational ambiguity, and that recovery may require role redesign and identity renegotiation as well as symptom reduction. Future research would benefit from examining how burnout becomes normalised within high-performance systems, how it comes to be recognised, and how recovery unfolds among coaches who remain in, leave, or later re-enter high-performance roles.

This study is based on a single autoethnographic case situated within a specific high-performance trampoline context. The analysis is based on retrospective reconstruction, which may be subject to selective recall and reinterpretation over time. To address this limitation, the study incorporated sustained reflexive documentation and ongoing critical dialogue with a critical friend, supporting the questioning and refinement of interpretations. The account also reflects the positionality of the first author. The findings provide contextually grounded and analytically rich insight into the relational and organisational processes through which burnout unfolds in high-performance sport.

## 5 Conclusion

This autoethnographic case study conceptualises coach burnout in high-performance trampoline coaching not as a discrete breakdown, but as a gradual and relational process unfolding over time. Across the Olympic cycle, expanding role conflict, political scrutiny, and responsibility without a corresponding capacity to reconcile competing expectations accumulated and became embodied in psychological and physical strain, leading not to clear recovery but to partial distancing and an ongoing reconfiguration of coaching identity.

By framing burnout as a temporal, relational, and interpretive experience, this study challenges linear accounts of breakdown and recovery. It further suggests that how burnout is experienced, interpreted, and responded to is inseparable from the relational and political conditions in which coaching is embedded.

These findings suggest that safeguarding coach wellbeing in high-performance sport requires attention not only to individual coping resources, but to the relational and organisational architectures that structure responsibility and legitimacy. Without corresponding structural protections, professional commitment may inadvertently become a mechanism through which vulnerability deepens. This study therefore invites further scholarly engagement with burnout as a situated, relational, and temporally unfolding phenomenon across diverse high-performance contexts.

## Data Availability

The original contributions presented in the study are included in the article/Supplementary Material, further inquiries can be directed to the corresponding author.
